# Index Catalogue of Library of the Surgeon-General’s Office

**Published:** 1884-11

**Authors:** 


### INDEX CATALOGUE OF THE LIBRARY OF THE SURGEON-GENERAL'S OFFICE, U. S. ARMY, VOL. V.

It is a notable feature of this book that the references upon the subject of the "heart" occupy one hundred pages, while those on the "head" claim fifty-nine pages. The titles of works on "hemorrhage" take up thirty-two pages, and those on "genitals" fill twenty-five pages, with twenty-one devoted to the "foot."

If their relative importance is to be inferred from the attention received by writers, this record becomes an interesting element in statistics.

Should medical men from all sections, who desire information on special subjects, be allowed to spend such time as may be requisite to consult the various authorities in this library, it would place the work of getting up this index upon a practical basis that it does not assume in its present technical aspect. The advantages afforded for general investigations of a literary nature by the great collection of books in the library connected with the British Museum might be extended to medical authors by the library of the Surgeon-General's office in the War Department of the United States, if facilities were granted for such examination of the list of books and pamphlets comprised in this Index and Catalogue.

This volume gives evidence of much care in its preparation, and though the labor is chiefly of a mere mechanical order, such as any systematic secretary can readily prepare, the work is overlooked by a most competent superintendent.

---

### ITEMS.

Dr. J. G. Hopkins, of this city, has moved to Thomasville, Ga.

An exchange says: "A widow shot herself in the oil regions the other day."

Dr. Fleetwood Churchill, the eldest son of the author of Churchill's Diseases of Women, died recently in Dublin, Ireland.

Dr. John H. Logan, of this city, has been elected to the chair of Chemistry in the Monroe Female College at Forsyth, Ga.

We had a pleasant call a few days since from Dr. Z. T. Daniel, of Washington, D. C. The Doctor is on a visit to his relatives in Georgia.

The Texas *Courier-Record* for September comes to us in a new dress, enlarged and very much improved in appearance. We wish for it the success it deserves.

The New Orleans *Medical and Surgical Journal* is now edited and published by an association of physicians, under the name of "The New Orleans Medical Publishing Association."

A movement was set on foot at the recent meeting of the Medical Society of Virginia to organize a Tri-State Medical Society, representing the States of Virginia, West Virginia and North Carolina

Dr. Wm. Osler, of Montreal, has been elected to the chair of Clinical Medicine in the University of Pennsylvania, rendered vacant by the transfer of Dr. Pepper to the chair of Theory and Practice of Medicine.

A student of the Medical Department of the University of New York recently committed suicide while riding in a cab. He had been for some time in a state of deep depression in consequence of the hopeless illness of his mother and sister.

The Atlanta Medical College opened October 9th with a large class. The introductory lecture was delivered by Dr. J. H. Logan. We are pleased to state that the hospital advantages of the College have been very greatly improved since the close of the last session.

Large numbers of dried and smoked lizards are imported by the Chinese physicians. They are used in cases of consumption and anæmia with considerable success. Their virtue seems to lie in the large amount of nitrogenous compounds and phosphates they contain.

A young medical student has offered himself to M. Pasteur as a subject for experiments with rabies which are to be made before a government commission. The student desires to be inoculated with the virus, and insists upon being given the preference in this distinction, expressing a heroic willingness to die, if need be, in the interest of science.

Speaking of the causes of failure in life, Tourgee says: "Trying to carry too big a load. I don't know about a professional man's failing if he works, keeps sober and sleeps at home. Lawyers, ministers, and doctors live on the sins of the people; and, of course grow fat under reasonable exertion, unless competition is too great. It requires real genius to fail in either of these walks of life."

On Saturday evening, September 20th, a complimentary dinner and reception were given by the numerous friends of Dr. Shoemaker, of Philadelphia, to him on his return from Europe, where he has been attending the meetings of the British Medical Association at Belfast, and those of the International Medical Congress at Copenhagen, as the representative of the National Medical Association of our land.

During the cholera season of 1873 a French physician in Nashville declared that he never lost a cholera patient. Shortly afterward, having gained some notoriety by this assertion, he was summoned to attend a man named White. The neighbors were anxious to see the result of the Frenchman's treatment, and whenever the doctor was seen leaving White's house numerous inquiries were propounded. "How is your patient, doctor?" was asked one morning. "Ze man is dead." "What! thought you never lost a case of cholera?" "Zis was ze first case I had. You not expect a man to lose one case before he get him. I cure ze man of ze cholera, but he die of ze weakness."—*Ex.*

Dr. R. O. Cotter, of this city, has resigned the place of assistant to the Chair of Eye, Ear and Throat Diseases in the Atlanta Medical College. He has gone on an extensive trip through the Southwest, and will probably locate in Texas. Dr. Cotter has been associated for the last four years with Dr. A. W. Calhoun, Professor of Diseases of the Eye, Ear and Throat in the Atlanta Medical College, and has made quite a reputation here. In the social world Dr. Cotter stands deservedly high. We regret to lose him. Dr. Cotter has been a constant contributor to THE JOURNAL, and we hope that he will continue his contributions from his new field.

